# The History and Capabilities of Herbal Simples

**Published:** 1890-07-05

**Authors:** W. T. Fernie


### The History and Capabilities of Herbal Simples.

By W. T. Fernie, M.D.

(*Continued from page* 180.)

### XIII.—THE CABBAGE.

"Now," as the walrus said in "Alice and the Looking Glass," "the time has come to talk of many things; of shoes, and ships, and sealing wax, of Cabbages and kings." So I will next proceed to discuss

### The Cabbage.

The cabbage is said to have first sprung from the tears of the famous Spartan lawgiver, Lycurgus. It began as the colewort, and was, according to Cato and Pliny, for six hundred years the only internal remedy used by the Romans. The Ionians had such a veneration for cabbages that they swore by them, just as the Egyptians did by the onion. With ourselves the wild cabbage growing on English sea cliffs is the true collet, or colewort, from which have sprung all our varieties of cabbage, cauliflowers, greens, broccoli, etc. As cultivated coleworts several of these came to us from Holland and Flanders. No vegetables were grown in England before the time of Henry VIII. In the thirteenth century it was the practice to salt vegetables because they were so scarce; and in the sixteenth century a cabbage from Holland was deemed a choice present. In 1595 the sum of twenty shillings was paid for six cabbages, and a few carrots at the port of Hull by the purveyor to the Clifford family. The generic name colewort is derived from, caulis, a stalk, and wourte, as applied to all kinds of herbs that "do serve for the potte." "Good worts" exclaims Falstaff, catching at Evan's faulty pronunciation of words, "Good worts! Good cabbages!" The term cabbage is from caba, a head, signifying a variety of colewort that forms a round head. The whole tribe of cabbages is named botanically, Brassicaceæ—"apo tou brassein,"—because they heat, or ferment. By natural order they are cruciferous plants. All cabbages contain much nitrogen, or vegetable albumen, with a considerable quantity of sulphur; hence they tend strongly to putrefaction, and when putrefied their smell is very offensive. When cut in pieces and pressed close in a tub with aromatic herbs and salt, so as to undergo an acescent fermentation, (which is arrested at that stage), cabbages form the German saurkraut. This is a favourite food and is strongly recommended against scurvy. The white cabbage is most putrescible, the red most emollient and pectoral. The juice of the red cabbage made into syrup, without any condiments, is useful in chronic coughs, and in bronchial asthma. The leaves of the common white cabbage when gently bruised and applied to blistered surfaces, will promote a free discharge, as also when laid next the skin in dropsy of the ankles. The sea cabbage—brassica oleracea—should be boiled in two waters, of which the first will be laxative, and the second, or thicker decoction, astringent. This was known to Hippocrates, who said "Jus caulis solvit, cujus substantia stringit." All the coleworts are called Crambe, from krambo, dry, because they drive away drunkenness. "There is," says an old author, "a natural enmitie between the colewort and the vine, which is such that the vine, if growing near unto it, withereth, and perisheth; yea, if wine be poured into the colewort while it is boiling it will not be any more boiled, and the colour thereof will be quite altered." In Kent and Sussex when a cabbage is cut and the stalk left in the ground to produce greens, the cottager cuts a X on the top of the upright stalk, and thus protects it against mischievous garden sprites and demons. An Irish cure for sore throat is to tie cabbage leaves round it; and the same remedy is employed in England with hot cabbage leaves for a swollen face. Thirty or forty years ago medical apprentices were taught the art of blood-letting by practising with a lancet on the veins of a cabbage leaf. No kale, cabbage, or other green vegetable is allowed to be served up at a Scotch wedding, because the colour green is regarded as unlucky in the land of cakes. The free presence of hydrogen and sulphur causes a very strong and unpleasant smell to pervade the house during the cooking of cabbages; nevertheless this sulphur makes them very salubrious. Among tailors to "cabbage" is a cant phrase which means to filch the cloth when cutting out for a customer. Arbuthnot says "your tailor instead of shreds, cabbages whole yards of cloth." The term seems to be derived from the French—*cabasser*—to put into a basket. Partridge and cabbage make a patrician dish; bacon and cabbage better suit the taste and the requirements of the rustic stomach.

